# Adapting narrative exposure therapy for Chinese earthquake survivors: a pilot randomised controlled feasibility study

**DOI:** 10.1186/s12888-014-0262-3

**Published:** 2014-10-03

**Authors:** Yinyin Zang, Nigel Hunt, Tom Cox

**Affiliations:** Division of Psychiatry and Applied Psychology, School of Medicine, University of Nottingham, Jubilee Campus, Wollaton Road, Nottingham, NG8 1BB UK; Center for the Treatment and Study of Anxiety, Department of Psychiatry School of Medicine, University of Pennsylvania, Philadelphia, PA 19104 USA; School of Business, Economics and Informatics, Birkbeck, University of London, Torrington Square, London, WC1 UK

**Keywords:** Chinese earthquake, Narrative exposure therapy, Posttraumatic growth, PTSD, Trauma

## Abstract

**Background:**

Narrative exposure therapy (NET) is a brief, manualised treatment for Posttraumatic Stress Disorder (PTSD). It has been shown to have therapeutic benefits for a wide range of individuals and settings. This study, following our previous work applying the original NET in earthquake survivors, aimed to revise NET to be adaptable for treating PTSD after a natural disaster.

**Methods:**

A randomised waiting-list controlled study was conducted with 30 adult participants with PTSD who were randomly allocated to NET (n = 10), revised NET (NET-R; n = 10) or a waiting list condition (WL; n = 10). Participants in NET and NET-R received treatment immediately; those in the WL condition received NET-R treatment after a waiting period. All groups were assessed on PTSD, general distress, anxiety, depression, social support, coping and posttraumatic change before and after treatment and three-month follow-up.

**Results:**

Compared with WL, both NET and NET-R groups showed significant reductions in PTSD and related symptoms. Significant increases were found in posttraumatic growth, active coping and perceived social support. The WL group showed similar improvements after treatment. Further reductions on PTSD symptoms were found at three months, showing that NET-R is as effective as the original NET in treating post-earthquake traumatic symptoms in adult Chinese earthquake survivors.

**Conclusions:**

NET-R is a feasible and cost-effective intervention for Chinese earthquake survivors. Further studies are needed to replicate these findings in other survivor populations, and with larger samples and over longer periods. This study highlighted the value of oral narrative approach, which is well-accepted and useful in the context of single natural disaster and lower- income area.

**Trial registration:**

Chinese Clinical Trial Registry: ChiCTR-TRC-12002931

## Background

People who are exposed to traumatic events have a profound need to make sense of them [1]. Enduring maladaptive responses characteristic of PTSD develop partly because the trauma has impacted negatively on autobiographical memory or narrative structure for the event [2,3], which hinder the individual’s meaning making process. There is an increasing body of empirical evidence for the efficacy of narrative treatments for traumatic disorders [4,5], for example, *Narrative Exposure Therapy* (NET) [6], and *Expressive Writing* [7]*.* Narrative approaches draw on normal human processes relating to story making and telling to help people recount the trauma with factual and emotional detail [8].

NET is a standardized short-term trauma-focused treatment approach developed to meet the needs of traumatised survivors of war and torture [9,10]. It is based on exposure therapy, cognitive behaviour therapy (CBT) and testimony therapy. In contrast to other exposure treatments, the participant do not identify a single traumatic event as a target in therapy. Instead, NET involves constructing a narrative that covers the participant’s entire life [11,12]. Cognitive processing models (e.g. [3]) suggest that PTSD symptoms are maintained through a distortion of explicit autobiographic memory about traumatic events and its detachment from the contents of implicit memory, which produces a fragmented narrative of the traumatic memories. Emotional processing theory [13,14] states that the habituation of emotional responses through exposure leads to a decrease in post-traumatic symptoms. NET stresses the importance of both approaches: the habituation of emotional responding to reminders of the traumatic event and the construction of a detailed narrative of the event and its consequences.

NET is a strict manualised treatment [9,10]. Sessions are usually 60–120 min in length and ideally occur in close succession. The person initially undergoes psychoeducation, then constructs a lifeline, with subsequent sessions dedicated to the narration of the person’s life, with particular focus on and attention to the traumatic events, which are narrated in great detail, ensuring emotional engagement with the memory. The aim is to integrate the generally fragmented, gap-filled reports of traumatic experience into a coherent narrative and to bring about the habituation of emotional responses to reminders of the traumatic event. Each session may focus on a single event, so people with multiple traumas need multiple sessions. Finally, the person and therapist have created a testimony of the person’s life from birth to the present day, with a detailed narration of the traumatic events.

A review of NET [15] showed it to be effective for those with PTSD following multiple traumatic events such as war or organised violence. Studies of NET in adults have consistently demonstrated its efficacy in treating individuals with PTSD and comorbid disorders living in a variety of low- and middle-income settings. Neuner and colleagues [11] studied Sudanese refugees in a Ugandan refugee camp and showed that NET was more effective than support counselling and one-session psychological education. Another randomized controlled trial study with 277 participants with Rwandan and Somali refugees in Uganda [12] demonstrated how both mental health professionals and lay counsellors can deliver NET, and indicates the effectiveness of NET and relatively lower dropout rates compared with flexible trauma counselling.

Although cognitive behaviour therapy (CBT) and Eye Movement Desensitization and Reprocessing (EMDR) have been shown to be effective in treating PTSD caused by a range of single traumatic event [16-18], they are still not sufficiently brief for use after large-scale disasters where there are large numbers of people who need assistance quickly, and there are likely to be few therapists available who are highly trained, as is required for CBT and EMDR. Being simple and relative easily trainable, NET is a good therapy for disaster victims with limited resources and low socioeconomic status. As oral narrative is common to all cultures, and previous studies have supported the effectiveness of NET in low and middle-income settings, NET is likely to be appropriate for disaster survivors in developing areas.

We conducted a study after the 2008 Sichuan earthquake which supported the effectiveness of NET in treating PTSD in Chinese earthquake survivors [19]. We reported issues in the process of therapy which suggest that NET could be further adapted and shortened for use after a single traumatic event. NET is originally designed for victims of multiple traumatic events, so the number of sessions may be reduced for survivors who have only experienced a single event. Participants in the previous study were interested in constructing their lifeline, but not in signing off their final written biography. This is likely because natural disasters do not have perpetrators, and so do not influence survivors’ dignity in the way that man-made war, torture, or violence does, so they had no need to regain it through the explicit human rights orientation of “testifying” [9]. Furthermore, the therapist anecdotally reported that most emotional resolution appeared to take place in the early sessions.

These issues raise a number of questions: (1) whether NET could be delivered in a shorter and more intensive format, (2) whether a reinforcement on the single traumatic event narration would lead to a better treatment effect, and (3) how well such intensive treatments are tolerated.

Intensive and short trauma-focused approaches such as CBT have been evaluated for treating PTSD. Ehlers and colleagues [20] found that an intensive 5–7 day CBT for PTSD is a feasible and promising alternative to weekly treatment. Basoglu and colleges [21] positively evaluated a single-session behavioural treatment for earthquake-related PTSD. All trauma-focused treatment protocols require the patient to confront their traumatic memories, but the methods of confrontation and their duration vary. It is not clear how well patients with PTSD would tolerate these procedures in a short and intensive treatment format.

In the present study, we made two revisions of the original NET on: (1) reinforcing the narration of a single traumatic event to strengthen the treatment effect; (2) cancelling the testimony sign-off to ease the therapist’s burden and enable them to treat more people in need following a large-scale disaster. It remains unknown whether these changes will influence the effects of other therapeutic elements such as the lifeline. Therefore, this study aims to explore the feasibility, acceptability and effectiveness of the revised NET (NET-R) for earthquake-related PTSD.

Many studies have shown that social support is important in the development and maintenance of PTSD in diverse trauma populations [22]. The ability of perceived social support to protect disaster victims’ health and mental health has been demonstrated repeatedly [23]. Coping style is also important to understand the psychological consequences of traumatic events [24]. Studies showed significant positive relations between coping efforts and symptoms (more coping, more distress) [24,25]. Data most consistently suggest that avoidance coping is problematic [23], but most people use different types of coping simultaneously, making it difficult to isolate their unique effects. Few studies have examined coping style in a longitudinal setting and little is known about the effect of exposure therapy on coping. In addition, people have reported perceived benefits following disaster [26]. Our previous study [19] measured social support, coping and posttraumatic growth pre and post treatment. It is found that the treatment could not change social support or coping in a short time, but could improve posttraumatic growth significantly and stably. Here, this study assessed the social support, coping and posttraumatic growth to investigate the effect of revised NET.

It is expected that an intensive version of NET would be adaptable for Chinese earthquake survivors, and would lead to same recovery effect over a shorter period of time than the original NET.

Specifically, it was hypothesised:

1. NET-R and NET would both significantly decrease symptoms of PTSD, depression, anxiety, and improve general mental health.
2. Both treatments would not improve perceived social support and coping in a short time.
3. Both treatments would lead to positive change.
4. Participants in the NET and NET-R groups would present with a preferable outcome at a 3-month follow-up.

## Methods

### Participants

The study used waiting list controlled randomization and took place between October 2010 and January 2011 (30–34 months after the earthquake) in Beichuan County. Beichuan was one of the areas nearly completely destroyed by the earthquake of 12th May, 2008. Almost all the buildings, including houses, working places, schools, and hospitals were destroyed. In Beichuan, 11522 people died and 9693 people were injured due to the earthquake [27]. Individuals experienced the Sichuan earthquake and reported PTSD symptoms were included in this study. They were initially screened by a door-to-door visit of a research team of 4 researchers led by the first author. If residents’ symptoms were considered to be probable PTSD, they were subsequently assessed in a face-to-face interview based on the PTSD Diagnostic Scale (PDS; [28]). Demographical information, previous traumatic experiences and features of earthquake exposure were also assessed in the interview. Question “what other traumatic events did you experience?” and PDS event list (Part A of the PDS) was used to assess participants’ previous traumatic events. Eligible participants were adults aged 18 or over who met the DSM-IV criteria of PTSD as measured by the PDS. Exclusion criteria included suicidal ideation or substance abuse, participation in another psychological treatment programme, and an inability to finish the treatment. Forty six residents were interviewed initially. Thirty people met the inclusion criteria and were recruited to participate in the study. All participants gave informed consent after receiving a full explanation of the study design, objectives and explicit information regarding what the study entailed. The study was carried out in compliance with the Helsinki Declaration and ethical approval was granted by the Ethical Committee of the University of Nottingham.

### Measures

Severity of PTSD symptoms was assessed using the *Impact of Event Scale-Revised* (IES-R; [29]). This instrument is a self-report measure comprising 22 items and three subscales (intrusion, hyperarousal and avoidance), and scored on a 5-point Likert scale from not at all (0) to extremely (4). Cronbach alpha for the three subscales of the Chinese IES-R have been reported as between .83-.89 [30], providing good evidence that the Chinese IES-R is a reliable and valid measure for assessing posttraumatic stress symptoms in a Chinese-speaking sample [31,32].

Depression and anxiety were assessed using the *Hospital Anxiety and Depression Scale* (HADS; [33]). This has also been translated into Chinese and widely used. In a sample of Chinese hospital in-patients, Cronbach’s alpha is .76 for depression and .79 for anxiety [34]. A score above 8 in both subscales was identified as probable anxiety or probable depression in the Chinese sample with the sensitivity of .74 and .78 respectively [34].

The Chinese version of the The *General Health Questionnaire-28* (GHQ-28; [35]) has been validated and adopted widely in China [36] and with a reported Cronbach alpha of .92 with a sample of Chinese earthquake victims [37].

The *Short Form of the Changes in Outlook Questionnaire* (CiQQ-S; [26]) was used to assess both positive and negative posttraumatic changes. The 10-item CiQQ-S consists of 5 items assessing positive changes, and 5 items assessing negative changes. Each item is answered on a 6-point scale ranging from strongly disagree (1) to strongly agree (6). The measure has been used in studies with a wide variety of participants following trauma and adversity [38]. The Cronbach alpha of Chinese version of CiOQ-S is .87 for positive change scale, and .82 for negative change scale [39].

The *Multidimensional Scale of Perceived Social Support* (MSPSS; [40]) was used to measure social support. The scale is designed to assess perceptions of the adequacy of social support from three different sources: family, friends, and significant others. It consists of 12 items; each item is scored using a 7-point Likert scale ranging from 1 (strongly disagree) to 7 (strongly agree). Adequate reliability and validity have been reported for a Chinese version [41], with a Cronbach alpha of .89.

Coping strategies were assessed with the widely used 28-item *Brief COPE* [42], which includes subscales that assess 14 different types of copings. Respondents were instructed to rate each item (1 = I have not been doing this at all to 4 = I have been doing this a lot) in relation to how they had “been coping with the stress during the past week”. The Brief Cope was validated in a sample of 168 community survivors of Hurricane Andrew. Cronbach alpha scores for the subscales ranged from .50 to .90. Factor analysis confirmed that the factor structure of the Brief Cope was similar to the full inventory [42]. The current study used a Chinese translation of Brief COPE, translated and back-translated by three psychologists (PhD level) who were proficient in both Chinese and English.

### Treatment programme

NET-R is modified based on the NET setting from the handbook [9] and issues raised in practice. The principle followed in the modification was to keep the effective elements of NET, but make it more practical and efficient in the context of single traumatic event. Table 1 shows the session settings of the original NET and NET-R.

**Table 1:** **Comparison of the treatment processes of the original NET and revised NET**

	**Original NET for PTSD after organised violence**	**Revised NET for PTSD after earthquake**
**Session 1**	● Introduction	● Introduction
	● Pre-treatment diagnostics	● Pre-treatment diagnostics
	● Psychoeducation	● Psychoeducation
**Session 2**	● Lifeline	● Starting the earthquake narration
	● Starting the narration beginning birth continuing through to the first traumatic event	
**Session 3 and subsequent sessions**	● Reading of the narrative collected in previous sessions. Continuing the narration of subsequent life and traumatic events	● Continuing the narration of the earthquake if needed
**The last session**	● Rereading and signing whole document	● Lifeline
		● Narrating from birth to current time, including reading of the earthquake narrative collected in previous sessions.
		● Reading the recorded final autobiography
**Frequency**	➢ Weekly or biweekly	➢ 1-2 days interval

NET-R was shortened to one week, and the interval between sessions was cut down to 1–2 days. The therapeutic process (e.g. narration correction, emotion habituation, etc.) was retained and followed, though the order was shifted to bring forward the earthquake narration to before the construction of the lifeline. Finally, the testimony sign-off was removed.

During the NET sessions, the participant, assisted by the counsellor, constructed a detailed chronological report of his/her own biography with a special focus on the traumatic experiences. In the NET-R condition, the participant first constructed a detailed earthquake narrative, and then completed the chronological autobiography with the assistance of the counsellor. In both conditions, the narrative was recorded by the counsellor and corrected with each subsequent reading. Participants were encouraged to relive emotions while reporting the events. In the last session, the participants in the NET group received a written report of the narrative, and the NET-R participants had the final reading of their autobiography without a written report.

### Procedure

**Figure 1:**
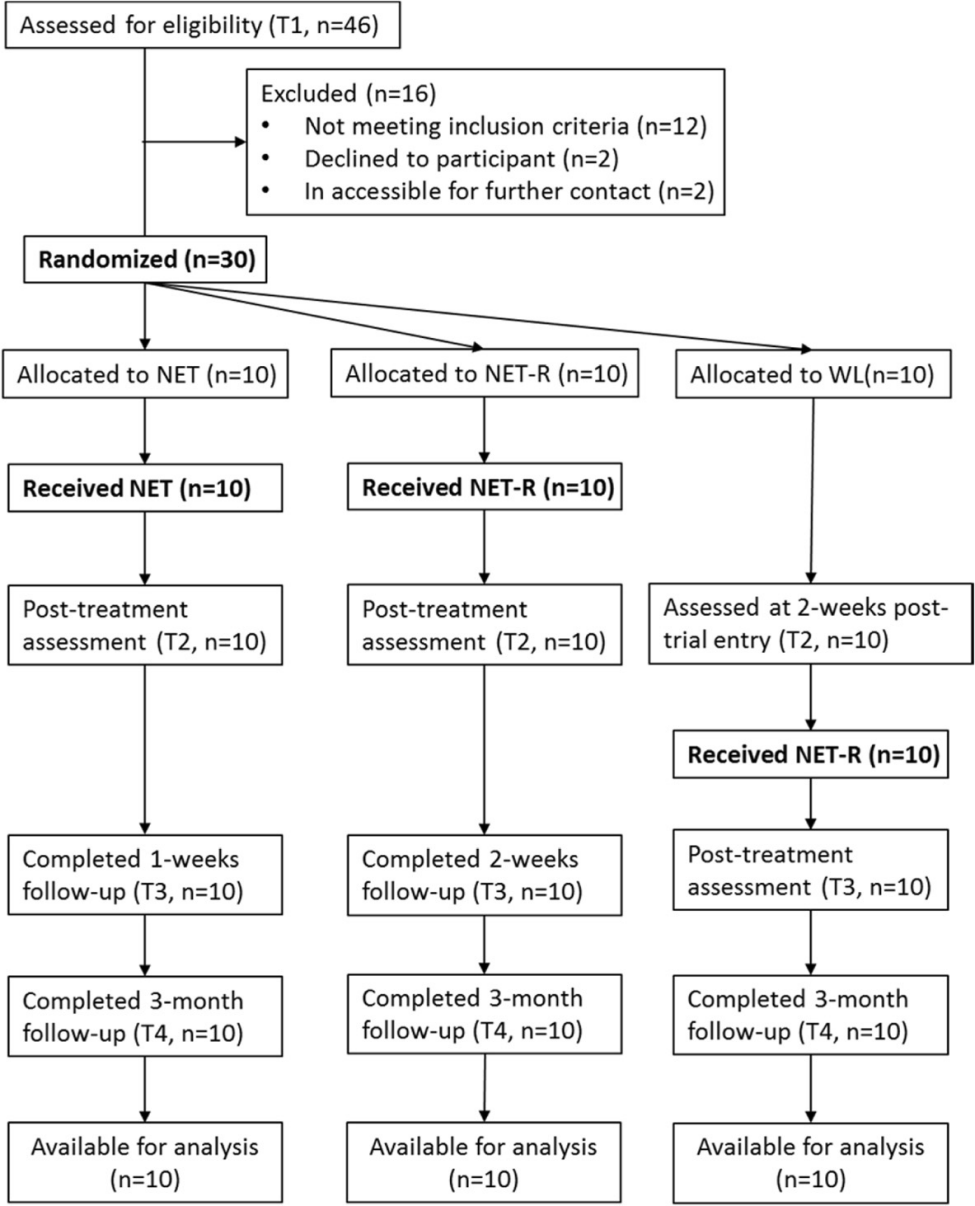
**CONSORT diagram showing the flow of participants through each group.**

Thirty participants were randomly allocated to either NET (n = 10), NET-R (n = 10) or a waiting list condition (WL; n = 10) by a computer-generated list of random numbers. Those in the NET and NET-R conditions received treatment immediately; those in the WL condition received the NET-R treatment after a three week waiting period. The assessment of screening process (T1) was used as the baseline. Those in the NET condition received 4 or more therapy sessions of 60–90 minutes each, which were administered about twice weekly for two weeks. The NET-R group received 3 or more therapy sessions of 60–120 minutes each, and the sessions were 1–2 days apart. Both NET and NET-R were assessed post treatment (T2), after another 1 week (for NET condition) or 2 (for NET-R condition) week (T3) and then after 3 months (T4) by using same scales. The WL controls were assessed 2 weeks after trial entry (waiting period) (T2), then given NET-R and assessed post treatment (T3) and finally assessed after 3 months (T4). Participants were informed that all scale items focused on the earthquake as the trauma event to make sure that the latent psychological variables were associated with exposure to the earthquake. Figure 1 presents the research and treatment schedules for the three conditions. There were no drop-outs, with all participants completing the entire course of treatment and follow-up.

Treatments for both the NET, NET-R and WL groups were carried out by the first author and one female psychological counsellor in the team. Both of them were native Chinese speakers with the Chinese national psychological counsellor certificate (Masters’ level) and were trained in the use of NET and NET-R based on the manual [9] and the modifications. Counsellors were closely supervised before beginning to work with clients. Case and personal supervision were maintained on a weekly basis. Treatment adherence was monitored by the direct observation of treatment sessions, by case discussions in supervision meetings, and by a review of the records and treatment protocols. The pre- and post- treatment assessments were carried out by a trained assessor not involved in the treatments and blind to the treatment conditions. The details of the condition were unknown to the assessor. The three month follow-up assessment was conducted by the first author over the telephone before the data analysis. All scales were administered orally by interview as most participants were illiterate or had problems reading (e.g., sight degeneration).

### Statistical analysis

Group differences in demographic data and pre-treatment measures were analysed by using chi-square tests and two-tailed t-tests. Pre- to post-treatment changes in questionnaire scores were analysed using univariate analyses of covariance (ANCOVAs), while controlling for pre-treatment scores. Within-group changes of each group from pre- to post- treatment were tested using paired t-tests. Hedge’s g was calculated as effect size for within- and between-group changes. The long-term treatment effects were analysed using repeated measures ANOVAs with the pre-test, post-test and follow-up scores and three groups. Pairwise differences were measured using paired t-tests with a Bonferroni correction. All analyses were performed in SPSS version 16.0.

## Results

### Treatment adherence

All participants constructed and completed a detailed chronological account of their own biography. The number of traumatic events they experienced is reported in Table 2. Most of participants did not experience other traumas except for the earthquake. Their reported previous traumatic experiences included difficult life conditions, family member’s terminal disease, or accidental injury. They did not report events such as violence, torture or persecution that described in previous NET studies of refugees. All Participants of NET condition spent no more than one session on narrating previous traumatic events, and then used two to three sessions focused on the single incident of the earthquake. They completed the treatment with four sessions in two weeks. Participants of the NET-R and WL conditions narrated their earthquake experience with two or three sessions, and spend no more than one session on narrating previous traumatic events. They finished the therapy within three or four sessions in one week. All participants completed the treatment. No major deviation from the study protocol occurred.

**Table 2:** **Sociodemographic characteristics of participants within the three treatment groups**

	**NET N = 10**	**NET-R N = 10**	**WL N = 10**	**Analysis**	
				***X*** ^***2***^	***p***
**Gender:**				2.22	0.33
Male	1	2	0		
Female	9	8	10		
**Marital status:**				2.65	0.62
Single	0	1	0		
Married	8	6	8		
Divorced or widowed	2	3	2		
**Education:**				1.25	0.54
Primary school or below	7	9	8		
Junior middle school	3	1	2		
**Income:**				2.24	0.70
No fixed income	4	6	7		
Below £100	2	2	1		
£100-£300	4	2	2		
**House damage**				3.36	0.17
Partially damaged	0	3	2		
Slightly damaged	10	7	8		
**Injured in the earthquake:**				1.36	0.51
Yes	4	2	2		
No	6	8	8		
**Bereavement**				5.16	0.08
Yes	8	3	6		
No	2	7	4		
**Number of traumatic events experienced besides the earthquake**				6.00	0.20
No	8	8	8		
Once	0	0	2		
2 or 3 times	2	2	0		
**Age**	M(SD)	M(SD)	M(SD)	*F*	*p*
	53.50 (1.24)	56.50 (1.47)	50.90 (1.23)	0.45	0.64

### Baseline data

The age range of the sample was 28 to 80 (53.63 ± 12.91). The socio demographic characteristics of the participants are described in Table 2. All of them were of low socio-economic status. With regard to earthquake exposure, all participants reported seeing someone seriously injured and death during the earthquake. Their own injury and house damage information are also reported in Table 2. There were no significant differences among the three groups regarding age, gender, education, marital status, income, injury, and house damage.

Table 3 shows the mean scores of scales, except for brief COPE, of three groups at each time point (T1, T2, T3, and T4). At baseline (T1), there was no significant difference among three groups.

**Table 3:** **Measures over time for NET, NET-R and WL groups**

**Measures**		**T1**		**T2**		**T3**		**T4**	
		***Mean***	***SD***	***Mean***	***SD***	***Mean***	***SD***	***Mean***	***SD***
**IES-R**	*NET*	50.90	10.65	17.00	6.72	16.10	6.85	12.70	9.59
	*NET-R*	52.10	9.06	16.90	4.93	16.50	4.81	12.70	6.48
	*WL*	56.80	10.91	54.70	10.81	16.60	5.13	13.80	6.63
**GHQ-28**	*NET*	9.90	8.80	2.70	3.23	2.40	3.03	1.60	2.59
	*NET-R*	15.80	7.33	1.30	2.06	1.70	2.31	0.60	1.26
	*WL*	14.40	5.62	14.30	6.15	0.80	1.48	0.50	0.97
**Anxiety**	*NET*	10.50	3.69	4.60	4.14	5.00	3.20	3.90	4.93
	*NET-R*	10.60	3.66	4.80	3.77	3.90	2.85	2.90	1.52
	*WL*	12.40	6.08	12.80	6.65	4.40	2.84	3.60	2.22
**Depression**	*NET*	11.10	6.08	4.50	4.12	4.00	3.89	3.10	4.18
	*NET-R*	10.40	4.81	4.00	3.97	2.80	1.75	2.40	1.84
	*WL*	10.50	5.60	10.40	6.47	2.20	1.75	2.40	1.84
**Positive changes**	*NET*	18.70	8.77	25.50	6.65	25.20	6.12	25.80	6.00
	*NET-R*	20.80	6.75	26.30	4.62	26.90	4.18	26.10	3.67
	*WL*	23.90	6.14	25.30	6.45	26.00	4.27	26.30	3.56
**Negative changes**	*NET*	17.70	7.09	10.80	4.59	10.20	4.66	8.70	4.40
	*NET-R*	21.30	6.40	10.90	3.11	10.70	3.62	9.80	2.90
	*WL*	15.10	6.05	14.20	5.87	7.20	2.35	6.40	1.90
**MSPSS**	*NET*	61.50	13.29	64.80	9.24	64.80	9.57	65.20	9.10
	*NET-R*	60.00	11.92	64.20	7.86	64.40	8.21	65.00	8.01
	*WL*	63.70	7.85	65.60	7.34	69.80	6.76	70.50	6.80

### Initial treatment outcome

The initial treatment outcome analyses are described in Table 4.

**Table 4:** **Results of outcome measures of T1 and T2** ^**△**^

**Measures**	**Group**	**Mean difference (T1-T2)**	**95% CI**	**Within-group**		**ES**	**Between-groups**		**ES (vs. WL)**	**Post-hoc** ^**a**^
				***df***	***t***		***df***	***F***		
**IES-R**	*NET*	33.90	(29.85 to 37.95)	9	18.93***	3.65	2,26	103.70***	4.01	1/3***
	*NET-R*	35.20	(26.47 to 43.93)	9	9.12***	4.62			4.31	2/3***
	*WL*	2.10	(0.54 to 3.66)	9	3.04*	0.19				
**GHQ-28**	*NET*	7.20	(2.00 to 12.35)	9	3.17*	1.04	2,26	40.05***	2.26	1/3***
	*NET-R*	14.50	(9.21 to 19.79)	9	6.20***	2.58			2.71	2/3***
	*WL*	0.10	(−1.23 to 1.42)	9	0.17	0.02				
**HADS anxiety**	*NET*	5.90	(3.55 to 8.25)	9	5.69***	1.44	2,26	10.16***	1.42	1/3***
	*NET-R*	5.80	(2.86 to 8.74)	9	4.47**	1.50			1.42	2/3***
	*WL*	−0.40	(−1.09 to 0.29)	9	−1.31	0.06				
**HADS depression**	*NET*	6.60	(3.98 to 9.22)	9	5.71***	1.22	2,26	14.57***	1.04	1/3**
	*NET-R*	6.40	(2.36 to 10.44)	9	3.59**	1.39			1.14	2/3**
	*WL*	−1.40	(−2.48 to −0.32)	9	−2.94*	0.02				
**CiOQ positive**	*NET*	−6.80	(10.90 to −2.70)	9	−3.75**	0.84	2,26	1.84	0.03	
	*NET-R*	−5.50	(−9.86 to −1.14)	9	−2.85*	0.91			0.17	
	*WL*	0.09	(−1.39 to 1.58)	9	0.17	0.21				
**CiOQ negative**	*NET*	6.90	(3.69 to10.11)	9	4.87**	1.11	2,26	9.18***	0.62	1/3*
	*NET-R*	10.40	(5.87 to 14.93)	9	5.20***	1.98			0.67	2/3**
	*WL*	0.90	(0.27 to1.53)	9	3.25*	0.14				
**MSPSS**	*NET*	−3.30	(−7.75 to 1.15)	9	−1.68	0.28	2,26	0.14	0.09	
	*NET-R*	−4.20	(−12.21 to 3.81)	9	−1.19	0.40			0.18	
	*WL*	−1.90	(−4.04 to 0.24)	9	−2.00	0.24				

^△^Means difference, 95% CI, paired t-test, within group effect sizes, ANCOVA analyses, and between group effect sizes are presented.

^*a*^ *1 = NET group; 2 = NET-R group; 3 = WL group.*

*p < 0.05;**p < 0.01;*** P < 0.001.

Paired t-tests revealed that all three groups experienced significant reductions on PTSD symptoms and negative change from T1 to T2. On the scales of GHQ-28, anxiety, and positive change, there were no significant within-group changes in the scores for WL group across its waiting period, but there were significant within-group changes on the scores of NET group and NET-R group. Significant score reductions of the NET and NET-R groups and a significant score increase of the WL group were found on depression. With regard to social support, no significant changes were found on the MSPSS scale for all three groups.

Univariate ANCOVAs on post- treatment scores controlling for pre- treatment scores revealed significant between-group effects for IES-R, GHQ-28, anxiety, depression and negative change. Following the treatment at the waiting period (T2), there were significant differences between the scores of all three groups on PTSD, GHQ-28, anxiety, depression and negative change. Post-hoc tests revealed that the NET and NET-R groups displayed lower scores than the WL group on above measures. No significant differences were observed between the two active treatment conditions.

Within- and between-group effect sizes for the outcome measures are also included in Table 4. From pre to post treatment, large (> = .80) within-group effect sizes were found for the NET and NET-R groups on PTSD, GHQ-28, anxiety and depression, positive changes and negative change; very small (<=.20) within-group effects were found for WL group on the PTSD, depression and negative change. Large between-group (NET vs. WL & NET-R vs. WL) effect sizes were found for PTSD, GHQ-28, anxiety and depression. Moderate (.50-.79) between-group effect sizes were found on the negative change.

### Three-month follow-up outcome

As the WL group received the treatment after T2, the scores of T2 for WL group were taken as their pre-test baseline. The pre-test, post-test and 3-month follow-up scores were analysed using repeated measures ANOVAs with three levels of time: pre-treatment (T1 for NET and NET-R; T2 for WL), post treatment (T2 for NET and NET-R; T3 for WL) and at 3-month follow-up (T4 for all three groups) and treatment group (NET, NET-R and WL) as between-subjects variable.

**Figure 2:**
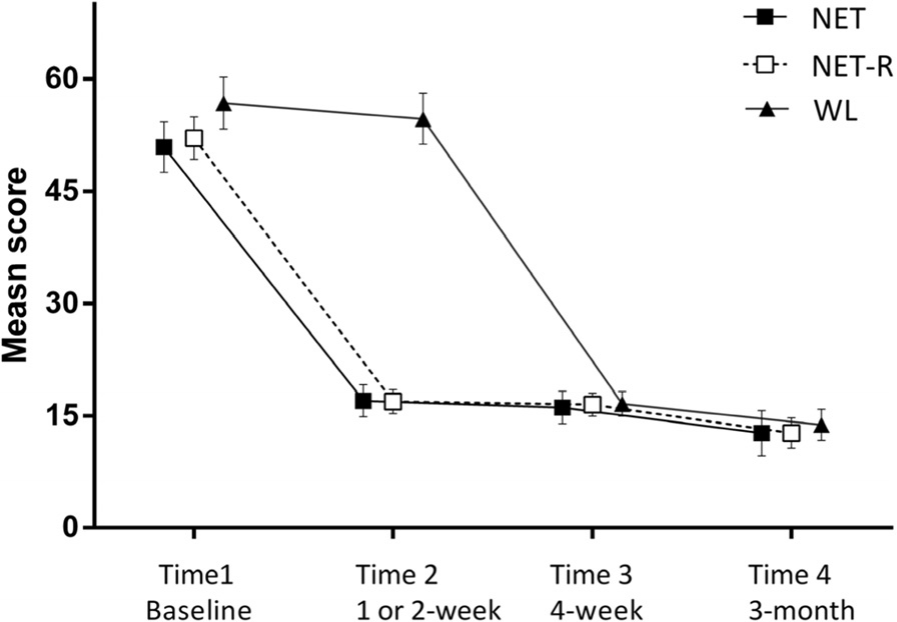
**Mean scores for IES-R of three groups.**

**Figure 3:**
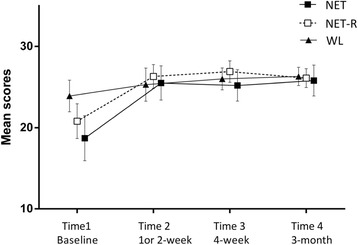
**Mean scores for positive changes of three groups.**

There were significant time effects for the measures of IES-R, *F* (2, 26) =172.89, *p* < 0.001; GHQ-28, *F* (2, 26) =51.09, *p* < 0.001; anxiety, *F* (2, 26) =26.00, *p* < 0.001, depression, *F* (2, 26) =36.91, *p* < 0.001; positive change, *F* (2, 26) =7.46, *p* < 0.01, negative change, *F* (2, 26) =44.08, *p* < 0.001; and MSPSS *F* (2, 26) =6.01, *p* < 0.01. There were no significant time × group interaction effects for any of the measures. Comparison of pre-, post-, and follow-up showed a significant reduction of scores after treatment on IES-R, GHQ-28, HADS, negative change (all *p* < 0.001), a significant increase on positive change (*p* < 0.01), and a trend towards higher on MSPSS (*p* = 0.05). From post-treatment to follow-up, a further significant reduction of scores was revealed in IES-R (*p* < 0.01) and negative change (*p* < 0.01). No significant differences of scores from post- to follow-up were found in GHQ-28, HADS, and MSPSS. These findings indicated that, for all three groups, overall PTSD symptoms across the three PTSD symptom clusters (intrusion, avoidance, and hyperarousal, see Figure 2), general distress, depression and anxiety, and negative change all decreased with treatment. The positive change of the CiOQ (Figure 3) and MSPSS increased significantly. The PTSD symptoms and negative change further decreased after treatments. The treatment effect sizes at 3 month follow-up of PTSD were 3.61 for NET condition and 4.79 for NET-R condition.

### Treatment effect on coping

Paired t-tests found significant within-group score changes in active coping, reframing, planning, religion and self-distraction between T1-T2 (see Table 5). Analyses revealed significant increases on active coping of NET-R group, on reframing of both NET and NET-R groups, on planning of NET group, on religion of WL group, and on self-distraction of NET-R group. Large (> = .80) within-group effect sizes were found for the NET on planning, for the NET-R on active, reframing and self-distraction. Moderate within-group effect sizes were found for the NET on planning, and a small effect size was found for the WL on religion.

**Table 5:** **-Significant within group changes between T1-T2**

**Coping**		**T1**		**T2**		**Within group**		**Effect size**
		***Mean***	***SD***	***Mean***	***SD***	**df**	***t***	
Active coping	NET-R	3.40	1.07	5.40	1.35	9	3.87**	1.57
Reframing	NET	4.40	1.90	5.50	2.01	9	2.91*	0.54
	NET-R	4.70	1.64	6.70	1.42	9	2.68*	1.25
Planning	NET	4.40	1.65	6.00	2.00	9	2.28*	0.84
Religion	WL	4.30	2.06	4.80	2.30	9	3.00*	0.22
Self-distraction	NET-R	6.90	1.20	7.90	0.32	9	3.87**	1.09

*p < 0.05;**p < 0.01; ***P < 0.001.

Univariate ANCOVAs on post- treatment scores controlling for pre- treatment scores revealed significant between-group effect on self-distracting, *F* (2, 26) =5.13; *p* < 0.05. The NET-R reported more distraction coping than the other two groups with large effect size (0.88) vs. NET group, and moderate effect size (0.47) vs. WL group.

Repeated measures ANOVAs of pre-, post- and follow-up scores showed significant time effects post-treatment for the measures of active coping, *F* (2, 26) =9.90, *p* < 0.001; reframing, *F* (2, 26) =11.99, *p* < 0.001; and planning, *F* (2, 26) =5.03, *p* < 0.05. There were no significant time × group interaction effects for any of the measures. Comparison of pre-, post-, and follow-up showed a significant reduction of scores after treatment in these three subscales, and the increases were stable at 3-month follow-up.

## Discussion

This study examined the feasibility and effectiveness of NET-R, an intensive, modified version of NET treatment for Chinese earthquake survivors, and compared NET-R with the original NET. The results indicated that both NET and NET-R were effective in treating survivors of the Sichuan earthquake, with no significant difference in effectiveness between these two therapies. Significant positive effects of both treatments were found across a number of psychological variables post treatment. Levels of reported symptoms of PTSD, anxiety, depression, general distress and negative change were significantly decreased. PTSD symptoms and negative change were further decreased at 3-month follow-up. The reductions of anxiety, depression and general mental health were stable for the 3 month follow up. NET and NET-R also showed effects on social support and coping. The positive change, perceived social support and some positive coping (active coping, reframing and planning) increased after the treatments and the changes were stable at follow up.

The improvements in posttraumatic symptom categories, anxiety, depression and general mental distress after the treatment replicated the results of our previous study [19], and supported the mechanism of emotional habituation elicited by exposure [13] and the efficiency of the narrative approach in dealing with the consequences traumatic events, such as intrusive memory fragmentation, avoidance, and reminders [3]. The comparable effectiveness of the NET-R with the original NET justified the modifications, indicating that the revision did not undermine the basis of NET, but made the therapeutic process more adaptable for earthquake survivors. The sizes of the treatment effect on PTSD symptoms at posttest (3.65 for NET & 4.62 for NET-R) were higher than previous NET studies with traumatized refugees populations, e.g. 0.6 in the study in Neuner et al. [11]. The treatment effect sizes on 3-month follow-up (3.61 for NET and 4.79 for NET-R) were also higher than the long-term follow-up effect sizes reported in other NET studies, e.g. 1.6-1.9 in Neuner et al. [11] and 1.4 in Neuner et al. [12]. Although the small sample size precludes drawing definitive conclusions about the efficacy of the NET or NET-R, the results are encouraging.

The further reductions of PTSD symptom and negative change from post-treatment to 3 month follow-up indicated that the treatments might function better in this study than previous NET studies. It also provided support for the predictive nature of negative change on PTSD. Previous work using the CiOQ found that negative change was the single best baseline predictor of six-month distress and well-being outcomes in a general sample of 1,657 respondents to an internet survey following the September 11, 2001 attacks in the United States [43]. This could link to the view that profound challenges to basic assumptions about the self, others, and the world is one of the most deleterious effects of traumatic experience [1]. Ehlers and Clark [3] suggested that a variety of idiosyncratic negative appraisals of the sequelae of the traumatic event can produce a sense of current threat and contribute to persistent PTSD. Individuals may interpret their symptoms as indications that they have permanently changed for the worse.

A significant improvement for post-treatment positive change, and the effects were stable at the 3 month follow-up. This result parallels with the previous study [19], and may link to meaning making produced by the narrative process [9,44]. In the present study, participants reported a positive appraisal of the central government’s rapid response to the earthquake. The social and political context could play a role in shaping survivors positive change after the earthquake. Participants of the present study (carried out 2.5 years after the earthquake) had been settled. The survivors, assisted by the government, had finished their house rebuilding. Some of them had already lived in their new houses, and the others were in the process of decorating. In this case, the improved stable environment probably also contributed to the efficacy of the treatments - the lessened livelihood burden could facilitate the treatment progress by avoiding the distraction of the secondary stressors. One study exploring the relationship between psychological harmony and satisfaction with government after this earthquake [45] found that satisfaction with local and central government was significantly related to the psychological harmony of survivors.

Contrary to previous NET study in the same population [19], perceived social support increased after the treatment. This may also link to the improved environment and settled residence of the participants in this study. The reduced sources of pressure may facilitate survivors’ reappraisal of the supportive efforts of others after treatments. This effect supports of the finding from previous studies that social support is important in the development and maintenance of PTSD [22,46], but implies that the relation could be moderated by the environmental factors and changes.

In terms of coping, the results indicated that treatments had a complex effect, but, in general, both NET and NET-R promoted some positive coping. Contrary to our expectation is that NET-R induced more self-distraction coping at the post-treatment, and the effect size was large. There are two possible explanations for this finding. First, NET-R may induce more earthquake related intrusive thoughts or stress that the participants choose to deal with by avoidance, but the decreased posttraumatic symptoms did not support this explanation. The second interpretation is that the increased distraction reflects enhanced adaptive response to the life stress. The exposure procedure of NET-R weakened the fear triggered effect of earthquake memory [13] and reduced intrusive thoughts of traumatic memory These changes might provide a positive experience of anxiety management, and participants might be able to learn from this experience and apply it to other stressful events. During the assessment interview, some participants explained that since thinking about the difficulties was useless, they might as well do other productive things. This may offer support for the second explanation. The increased distraction only was present for the NET-R group. The forward shift and reinforcement of the earthquake narrative in NET-R might better benefit participants through symptoms reduction in the early phase of the therapy. The improvement in positive coping (active coping, reframing and planning) of both NET and NET-R conditions may occur because successfully solving psychological distress enhanced participants’ sense of control, so they might start to respond positively to other solvable difficulties in their lives. Previous NET studies in victims of organised violence did not assess coping, but these findings suggest that a short term intervention could have a subtle and indirect effect on coping by reducing distress.

The lack of dropout is in line with other NET studies [11,19,47]. Most participants informally reported better sleep and were relieved after treatment. In addition, participants were mostly older and had low socioeconomic status. They found the therapeutic approach of narrative was acceptable and comfortable. The use of narration in NET, which is the approach we all use in daily life, is one of the reasons why we used this approach. It has high face validity, it does not intimidate people, and is appropriate for less educated people. The participants were talkative; most could initiate their narrative about the earthquake smoothly. Only two female participants; one who lost her daughter in the earthquake, and the other’s husband lost an arm, were reluctant to recall the earthquake memory at the outset. Their avoidance was understandable as the loss still had negative effects on their lives. This may suggest that practitioners should adjust the sessions according to the survivors’ individual experiences and current psychological condition. Furthermore, for the current sample, the treatment was aimed at people with only one incoherent, fragmented and traumatic memory of the earthquake; thus early assignment could take effect rapidly. Participants’ previous trauma experiences need to be clarified in the diagnostic interview before application of NET-R.

The results also support extending NET outside of situations where testimony may be required to other situations where people may benefit from developing narratives. Participants in the NET group were not interested in signing off their final written biography. Several explained that because they felt much better, there was no need to keep a written document. Also, as most of the participants were older and with low socioeconomic status, they did not read regularly. In the NET-R group, no participant asked for a written testimony at the ending session. This supported the hypothesis that as there are no perpetrators, participants do not think their human rights and dignity were prejudiced. However, a testifying document can also serve a function with some situation under natural disaster traumas. For example, it is useful when survivors need to take a stance against the governments, or on some conflicting social or societal issues. Again, the result indicated the flexible usage of NET in different situations or different populations. Both NET and NET-R retained the lifeline in the treatment. From the feedback of the consellors and participants, the lifeline is useful in establishing the therapeutic relationship and in providing an indication of the number of sessions that may be necessary to address all traumatic events.

The study replicated and extended the findings of previous NET studies. It investigated and compared the effects of a modified NET and original NET in a Chinese setting for earthquake-related PTSD. It provides evidence for the effective treatment elements of Western developed approach in the Chinese population. Although the efficacy of NET has already been shown across cultures in Europe, Africa and Asia [11,12,48], the psychosocial environment in this study was different from that of previous work which has largely focused on people affected by war and torture. Nevertheless, it demonstrated that modifications can be made to make the NET function more effectively. Having fewer sessions means that more people can be treated.

The main limitation of the study is the sample size and the lack of a longer term follow up. The size of the sample is relatively small as the study aimed to test the effectiveness and feasibility of a newly modified treatment, and practical considerations meant that a longer term follow-up was impractical in this disaster area. There were much more women than men in this study, because most men were out for work in the day time after the earthquake. However, the utility of NET-R in such circumstances was demonstrated.

## Conclusions

In conclusion, NET-R is a feasible and cost-effective intervention for Chinese earthquake survivors. Further studies are needed to replicate these findings in other survivor populations, and with larger samples and over longer periods. The oral narrative approach is useful in the context of single natural disaster. Cost- and time-effective treatments are particularly important after large-scale disasters, which often overwhelm the national mental health care resources of the affected countries. The relatively easily captured procedure and cross-culturally acceptable format of NET-R may facilitate its wider delivery after major disasters, and with people of low socio economic status. Future research to explore the most effective ways of training people to administer it is warranted.

## Abbreviations

NET: Narrative exposure therapy
NET-R: Revised narrative exposure therapy PTSD, Post traumatic stress disorder
IES-R: Impact of event scale-revised
HADS: Hospital anxiety and depression scale
CiOQ: Changes in outlook questionnaire
GHQ-28: The general health questionnaire-28
MSPSS: The multidimensional scale of perceived social support
WL: Waiting-list control
